# Do Elderly Lung Cancer Patients Aged ≥75 Years Benefit from Immune Checkpoint Inhibitors?

**DOI:** 10.3390/cancers12071995

**Published:** 2020-07-21

**Authors:** Nagio Takigawa, Nobuaki Ochi, Nozomu Nakagawa, Yasunari Nagasaki, Masataka Taoka, Naruhiko Ichiyama, Ayaka Mimura, Hidekazu Nakanishi, Hiroyuki Kohara, Hiromichi Yamane

**Affiliations:** General Internal Medicine 4, Kawasaki Medical School, Okayama 700-8505, Japan; placidus.aura@gmail.com (N.O.); nznaka0717@gmail.com (N.N.); yasunari1938@gmail.com (Y.N.); me422056@yahoo.co.jp (M.T.); narunba0421@gmail.com (N.I.); 2326ayaka3649@gmail.com (A.M.); nakanisi@med.kawasaki-m.ac.jp (H.N.); hkohara@med.kawasaki-m.ac.jp (H.K.); hiromichi.ya@gmail.com (H.Y.)

**Keywords:** elderly, immune checkpoint inhibitor, lung cancer

## Abstract

Lung cancer patients ≥75 years represent nearly 40% of all lung cancer patients and continue to increase. If elderly patients have a good performance status and adequate organ function, they can be treated the same as non-elderly patients. However, few comparative studies limited to elderly patients (≥75 years) have been conducted. We review the evidence on using immune check inhibitors for the treatment of elderly patients (≥75 years old) with advanced non-small cell lung cancer. Prospective randomized or non-randomized, retrospective, registrational, insurance-based, and community-based studies have shown that elderly (≥75 years) and non-elderly patients are similarly treated with immune check inhibitors effectively and safely. However, such analyses have not shown that immune check inhibitors are significantly more effective than chemotherapy alone. In addition, patient selection might be critically performed to administer immune check inhibitors in the elderly because they are more likely to have a poor performance status with comorbidities, which lead to little benefit, even in non-elderly patients. There is a need for more evidence showing the benefit of immune check inhibitors in non-small cell lung cancer patients ≥75 years.

## 1. Introduction

Before the era of molecular-targeted agents and immune checkpoint inhibitors (ICIs) such as a programmed cell death protein 1 (PD-1) antibody, the age of eligibility of elderly patients with advanced non-small cell lung cancer (NSCLC) in phase III randomized trials was often defined as 65 or 70 years [1], and only 30–50% of patients enrolled in these trials were aged ≥75 years [1]. In addition, there have been few recent studies on molecular-targeted agents and ICIs in patients ≥75 years of age [2].

The median age at diagnosis of lung cancer was 71 years old and patients ≥75 years old was 36.3% according to Surveillance, Epidemiology, and End Results (SEER) Cancer Statistics Review in 2013–2017 [3]. In addition, the median age at nivolumab or pembrolizumab initiation in real-world metastatic NSCLC patients was 69 years; 27% were aged 75 or older [4]. The Japanese guidelines for the diagnosis and treatment of lung cancer define elderly lung cancer patients as ≥75 years [5]. In 2015, the lung cancer incidence was highest in males aged 70–74 years, followed by males aged 75–79 years, and in females aged ≥ 85 years, followed by females aged 70–74 years [6]. Therefore, a cutoff age of 75 years is considered reasonable for distinguishing elderly from non-elderly groups among lung cancer patients.

Ferrara et al. reviewed the relationship of immunotherapy resistance with immunosenescence, which was probably linked to the continuous and progressive remodeling of immune functions with ageing [7]. The loss of senescence markers such as CD27 and CD28 or the expression of Tim-3 and CD57 on T cells was associated with resistance to ICIs [8]. Immunosenescence may be related to a decreased response to ICIs or to an increased risk of adverse events, especially in elderly NSCLC patients (≥75 years) [7].

If elderly patients have a good performance status (PS) and adequate organ function, they can be treated the same as non-elderly patients. However, few comparative studies limited to elderly patients (≥75 years) have been conducted. Even in a recent review of elderly NSCLC patients, the cut-off age was 65 or 70 years [9]. Although there was no upper age limit in the eligibility criteria for most recent prospective studies, the extent to which the results derived from non-elderly patients can be extrapolated to elderly patients is unknown. Here, we review the evidence on using ICIs for the treatment of elderly patients (≥75 years old) with advanced lung cancer, especially NSCLC.

## 2. Standard Anticancer Drug Therapies for Elderly Patients with Advanced NSCLC

Patients with advanced NSCLC with abnormalities in the epidermal growth factor receptor, anaplastic lymphoma kinase fusion, B-Raf proto-oncogene, ROS proto-oncogene 1 fusion, or neurotrophic tropomyosin receptor kinase fusion gene are recommended treatment with appropriate molecular-targeted drugs, which have also been used in the elderly [9]. In addition to these markers, it is important to evaluate the immunohistochemical expression of programmed death ligand 1 (PD-L1), as PD-L1 expression is a confirmed predictive factor for the treatment outcome of ICI monotherapy in NSCLC patients. Until several years ago, a single third-generation cytotoxic anticancer drug (docetaxel, vinorelbine, or gemcitabine) was recommended for NSCLC [9]. Recently, the results of a phase 3 study of carboplatin plus pemetrexed followed by maintenance pemetrexed compared with docetaxel monotherapy in non-squamous NSCLC patients ≥75 years old was reported [10]. Non-inferiority in survival time and superiority in progression-free survival were found in the drug combination group, and the regimen has become a standard treatment.

There is no randomized study comparing ICIs with chemotherapy in elderly NSCLC patients. If PS is 0 or 1 without severe comorbidities, pembrolizumab monotherapy may be used for PD-L1-positive cases [11,12]. In addition, regardless of PD-L1 expression, platinum combination therapy plus ICI is the standard treatment for non-elderly patients with a PS of 0 to 1 [13,14,15,16], but its usefulness in the elderly is not clear. There may be additional value in using ICI for carboplatin plus pemetrexed or carboplatin plus (nab-) paclitaxel combinations in this population. Later, we will review the use of ICI with or without chemotherapy for the treatment of advanced NSCLC patients ≥75 years old.

## 3. Standard Second-Line Treatment for Elderly Patients with NSCLC

There have been few randomized studies on second-line treatments for elderly patients (≥75 years old) [17,18]. When pembrolizumab is administered as a first-line treatment for PD-L1-positive NSCLC, a cytotoxic anticancer drug is used as a second-line treatment. In patients who do not receive pembrolizumab in first-line therapy, ICI such as pembrolizumab, nivolumab, or atezolizumab are candidates for second-line therapy. PD-L1 positivity (≥1%) is an essential criterion for the use of pembrolizumab, but nivolumab and atezolizumab can be used irrespective of PD-L1 expression [19].

## 4. Initial Treatment for Extensive-Stage Small-Cell Lung Cancer

The IMpower133 and CASPIAN phase 3 studies showed the efficacy of adding ICIs (atezolizumab or durvalumab) to first-line treatments comprising platinum and etoposide in patients with extensive-stage small cell lung cancer [20,21]. In the IMpower133 study, only 19 patients (9.5%) were ≥75 years of age in the carboplatin–etoposide–atezolizumab group and 22 (10.9%) in the carboplatin–etoposide–placebo group. In the CASPIAN study, the median age (interquartile range) was 62 (58–68) years in the durvalumab–etoposide plus cisplatin or carboplatin arm and 63 years (57–68) in the etoposide plus cisplatin or carboplatin arm. KEYNOTE-604 study comparing pembrolizumab plus etoposide and platinum with placebo plus etoposide and platinum for patients with previously untreated extensive-stage small cell lung cancer was also recently reported. Median age (range) was 64 (24–81) in the former arm and 65 (37–83) in the latter arm [22]. The numbers of patients ≥75 years old in both studies were not stated; thus, the efficacy and safety of ICIs in patients ≥75 years old with extensive-stage small cell lung cancer remain unknown.

## 5. Efficacy of ICIs in Elderly NSCLC Patients (≥75 years) in Phase 3 Studies

The hazard ratios (HRs) for overall survival (OS) in patients <75 and ≥75 years of age with advanced NSCLC in phase III studies are shown in Table 1.

In the KEYNOTE-024 and KEYNOTE-042 studies, which compared pembrolizumab monotherapy with chemotherapy in the first-line setting, a sub-analysis of patients ≥75 years old was not initially reported [11,12]; however, recently, a pooled analysis of patients ≥75 years old in those trials was published [23]. The HR (95% confidence interval [CI]) for OS in the KEYNOTE-024 study was 0.64 (0.42–0.97) in patients <75 years and 0.49 (0.17–1.39) in those ≥75 years. The HR for OS in the KEYNOTE-042 trial was 0.79 (0.68–0.92) in patients <75 years and 0.89 (0.59–1.35) in those ≥75 years. No significant difference was observed in the sub-analysis, but the favorable trend observed in the pembrolizumab group was maintained.

In the CheckMate 017 and CheckMate 057 studies, nivolumab was found to prolong OS compared with docetaxel as salvage therapy in patients with squamous cell carcinoma or non-squamous cell carcinoma [24,25]. CheckMate 017 showed significant differences in HRs (95% CI) of 0.52 (0.35–0.75) and 0.56 (0.34–0.91) in patients < 65 years and 65–74 years, respectively. The HR (95% CI) in patients ≥75 years was 1.85 (0.76–4.51), indicating a favorable therapeutic effect in the docetaxel group, but this effect was considered to be skewed by the small number of cases (29 patients). The HR in patients ≥75 years in the CheckMate 057 study was 0.90 (0.43–1.87), indicating similar effectiveness in both arms, although the sample size was small (43 patients). CheckMate 227 was a phase 3 study comparing nivolumab plus ipilimumab with chemotherapy for first-line treatment of advanced NSCLC [26]. An OS benefit was observed irrespective of PD-L1 expression level. The HRs (95% CI) were 0.70 (0.58–0.85), 0.76 (0.61–0.95), and 0.84 (0.55–1.29) in patients <65 years, ≥65 to <75, and ≥75 years old, respectively. The efficacy of combined ICIs in elderly patients (≥75 years) remains unknown.

KEYNOTE-010 is a phase 3 trial of salvage therapy consisting of docetaxel versus pembrolizumab (2 or 10 mg/kg) for the treatment of advanced NSCLC [27]. The HRs for OS (95% CI) in patients <75 and ≥75 years were 0.64 (0.55–0.75) and 0.72 (0.43–1.21), respectively. The cases (*n* = 90) ≥75 years old constituted <10% of those <75 years old (*n* = 943). Thus, the sub-analysis suggested efficacy of pembrolizumab in elderly patients with chemotherapy-refractory NSCLC. KEYNOTE-024 was a phase 3 study comparing pembrolizumab (200 mg Q3W) with platinum-containing chemotherapy in patients with treatment-naive advanced NSCLC with high PD-L1 expression (≥50%) [11]. The HRs (95% CI) for OS were 0.64 (0.42–0.97) and 0.49 (0.17–1.39) in patients aged <75 (*n* = 260) and ≥75 (*n* = 45) years, respectively [23]. Although the number of elderly patients was small, the HR of 0.49 was good, suggesting that elderly NSCLC patients with high PD-L1 expression may benefit from pembrolizumab monotherapy. Although the study design of KEYNOTE-042 was similar to that of KEYNOTE-024, the eligibility of PD-L1 expression was ≥1% and ≥50%, respectively [11,12]. The HRs (95% CI) were 0.79 (0.68–0.92) in patients <75 years (*n* = 1145) and 0.89 (0.59–1.35) in those ≥75 years (*n* = 129) [23]. Solely based on the HRs (0.49 in KEYNOTE-024 and 0.89 in KEYNOTE-042), elderly patients ≥75 years with 1–49% PD-L1 expression may not benefit from pembrolizumab monotherapy. Although the pooled analysis included first-line and salvage therapies, pembrolizumab monotherapy tended to improve OS compared with chemotherapy in patients aged ≥75 years (median OS (mOS): 15.7 vs. 11.7 months, respectively; HR: 0.76; 95% CI: 0.56–1.02), especially those with ≥50% PD-L1 expression (mOS: 23.1 vs. 8.3 months, respectively; HR: 0.40; 95% CI: 0.25–0.64) [23].

**Figure 1:**
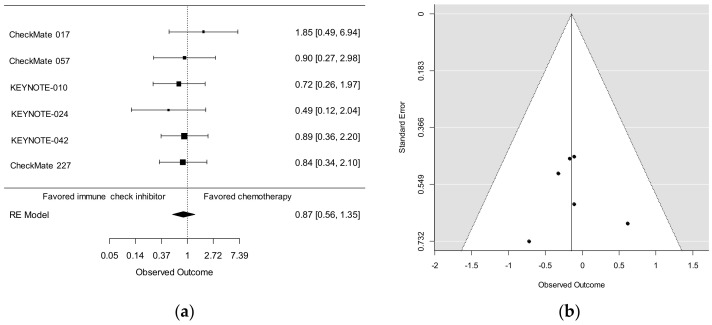
The meta-analysis was conducted using a random effects model, which weighed studies using the restricted maximum likelihood method. Studies were combined by pooling the hazard ratios (log scale) and corresponding standard errors. No significant difference was observed between immune checkpoint inhibitor therapy and chemotherapy (**a**). Funnel plots of the six studies revealed little publication bias (**b**). The package ‘metafor’ in the R Statistical Platform, v3.5.1 (R Foundation, Vienna, Austria), was used for the analysis.

Here, we performed a meta-analysis of the six previous studies that compared ICIs (monotherapy with nivolumab or pembrolizumab in five studies; doublets with nivolumab and ipilimumab in one study) with chemotherapy, irrespective of the line of treatment (first or second). The HR (95% CI) for OS was 0.87 (0.56–1.35), and the efficacy of ICIs in NSCLC patients ≥75 years was not significant (Figure 1a). Funnel plots of the six studies revealed little publication bias (Figure 1b). According to a meta-analysis in NSCLC patients ≥75 years who participated in four randomized studies (CheckMate 057, KEYNOTE-010, OAK, or POPLAR) [25,27,28,29], mOS in patients receiving PD-1/PD-L1 blocking antibodies versus docetaxel was 14.7 versus 9.5 months [30]. The HR (95% CI) of 0.81 (0.58-1.13) was similar to that in our analysis. Because the numbers of patients ≥75 years were not reported in the OAK and POPLAR studies [28,29], we could not include their data in our meta-analysis.

The Impower150 [16] and Impower131 [31] studies also compared ICIs plus chemotherapy with chemotherapy and reported HRs for OS in four age groups (<65, ≥65 to <75, ≥75 to <85, and ≥ 85 years). The results are summarized in Table 1. In IMpower150, the mOS was longer in the atezolizumab + bevacizumab + carboplatin + paclitaxel arm than in the bevacizumab + carboplatin + paclitaxel arm (19.2 vs. 14.7 months; HR 0.78; 95% CI, 0.64–0.96). The HR in patients 75–84 years old was 0.78 (0.50–1.76), which did not indicate a significant OS benefit in this subgroup [16,32]. The mOS in the IMpower131 trial was 14.2 months in the atezolizumab + carboplatin + nab-paclitaxel arm and 13.5 months in the carboplatin + nab-paclitaxel arm (HR: 0.88; 95% CI: 0.73–1.05) [31]. The HR (0.74 [95% CI: 0.45–1.23]) in patients aged 75–84 years old was also not significant. Thus, there is little evidence of the efficacy of ICIs in combination with chemotherapy for advanced treatment-naive patients ≥75 years old.

## 6. Efficacy of ICIs for Elderly NSCLC Patients in Non-Randomized Studies

Non-randomized studies of elderly NSCLC patients treated with ICI monotherapy are summarized in Table 2. 

CheckMate 153 [33] prospectively examined the safety and efficacy of nivolumab in patients with advanced NSCLC including patients aged ≥70 years with a poor PS, who are typically under-represented or excluded from randomized studies. The mOS was comparable in the overall population (9.1 months, *n* = 1426) and in patients ≥70 years (10.3 months, *n* = 556). CheckMate 171 [34], which had a similar design to CheckMate 153, only included patients with squamous NSCLC. The OS and response rate (RR) corresponding to the entire population (*n* = 811), patients ≥70 years (*n* = 278), and those ≥75 years (*n* = 125) were 10.0 months and 11.0%, 10.0 months and 12.6%, and 11.2 months and 13.6%, respectively. Thus, nivolumab for advanced, relapsed squamous NSCLC seems to be similarly effective between elderly and non-elderly patients.

In an Italian cohort of an expanded access program (EAP) using nivolumab for squamous NSCLC patients, 34% (*n* = 126), 47% (*n* = 175), and 19% (*n* = 70) of patients were aged <65, 65 to <75, and ≥75 years, respectively [35]. Although the RRs were similar (18%, 19% and 18%, respectively), the mOS was lower in patients ≥75 years (5.8 months) compared with those <65 years old (8.6 months), those 65 to <75 years old (8.0 months), and the overall population (7.9 months). An Italian cohort of EAP using nivolumab for non-squamous NSCLC has also been published [36]. The outcomes of the patients (total population, *n* = 1585; ≥70 years, *n* = 522; ≥75 years, *n* = 232) were similar with respect to RRs (18%, 21%, and 25%, respectively) and mOS (11.3, 11.5, and 12.0 months, respectively). In another Italian retrospective study, all consecutive advanced NSCLC patients using ICIs (anti-PD-1, *n* = 205; anti-PD-L1, *n* = 77; anti-CTLA4 or combination ICI therapy, *n* = 8) between April 2013 and March 2019 were analyzed [37]. The numbers of patients aged <70, 70–79, and ≥80 years were 180, 94, and 16, respectively; the RRs (21.5%, 22.3%, and 18.8%, respectively; *p* = 0.95) and mOS (9.1, 11.3, and 9.6 months, respectively; *p* = 0.52) were similar across age groups.

Using the SEER-Medicare database, Youn et al. [38] identified 1256 patients aged ≥65 years who had NSCLC between 2002 and 2015 and initiated nivolumab or pembrolizumab in 2016. These patients had stage I (*n* = 229), stage II (*n* = 75), stage III (*n* = 417), or stage IV (*n* = 535) cancer and had received no previous systemic therapy (*n* = 102). Multivariate survival analysis showed no difference in the OS according to age, with HRs (95% CI) of 1.00 (0.86–1.16) and 0.88 (0.67–1.16) for patients aged 75–84 (*n* = 545) and ≥85 (*n* = 106) years, respectively, when the HR for death was set to 1 in patients aged 65–74 years.

In the National Dutch NVALT Registry, 2302 patients with metastatic NSCLC treated with nivolumab, pembrolizumab, atezolizumab, or durvalumab received ICIs as first-line (*n* = 131), second-line (*n* = 1713), or third-line or higher (*n* = 458) therapy [39]. All patients had stage IV cancer, and their mean age was 63 years (range: 28–88). The mOS (95% CI) was 12.3 months (11.3–13.3) in patients aged ≤75 years (*n* = 2095) and 13.7 months (12.3–19.9) in patients aged >75 years (*n* = 207), and the HR (95% CI) was 0.84 (0.66–1.08) (*p* = 0.17).

Yamaguchi et al. [40] retrospectively evaluated the efficacy of subsequent-line nivolumab or pembrolizumab monotherapy in elderly Japanese patients (aged ≥75 years) with a median age of 77 years (range, 75–87). The mOS was 16.0 months (95% CI: 12.1–19.8) in all patients (*n* = 131). There was no significant difference in the mOS between patients 75–79 years old (13.1 months) and those ≥80 years old (18.9 months) (HR: 1.44; 95% CI: 0.83–2.68; *p* = 0.2). Thus, ICIs seemed to be as effective in elderly NSCLC patients ≥75 years old as in those <75 years old in prospective or retrospective non-randomized studies in EAP, SEER, and registry cohorts.

## 7. Comparison of Adverse Events Between Elderly and Non-Elderly Patients Treated with ICI Monotherapy

ICIs such as nivolumab and pembrolizumab are usually associated with fewer treatment-related adverse events (TRAEs) compared with chemotherapy in randomized studies [11,12,24,25,27]. However, few studies have compared TRAEs in NSCLC patients aged ≥75 or <75 years treated with ICIs. A pooled analysis of KEYNOTE-010, KEYNOTE-024, and KEYNOTE-042 studies showed that TRAEs seemed to slightly increase in patients ≥75 years compared with those aged <75 years, although statistical analysis was not performed [23]. Any-grade TRAEs were observed in 102 patients (68.5%), and grade ≥3 TRAEs were observed in 36 (24.2%) of 149 patients ≥75 years old. Meanwhile, any-grade TRAEs were also observed in 862 (65.2%) and grade ≥3 TRAEs in 224 (16.9%) of 1323 patients <75 years old.

Any-grade and/or grade 3/4 TRAEs in the elderly (including ≥75 or ≥80 years old) versus non-elderly NSCLC patients treated with ICIs in non-randomized studies are shown in Table 3.

Any-grade TRAEs caused by nivolumab were reported in 32%, 28%, 29%, and 29% of non-squamous cell NSCLC patients <65 years (*n* = 126), 65 to <75 years (*n* = 175), ≥75 years (*n* = 70), and in the overall population (*n* = 371), respectively [35]. Grade 3/4 TRAEs were observed in 3%, 9%, 3%, and 6% of patients <65 years, 65 to <75 years, ≥75 years, and in the overall population, respectively. The frequency of any-grade and grade 3/4 TRAEs caused by nivolumab were similar: 33% and 6% in all squamous NSCLC patients (*n* = 1585), 33% and 7% in those ≥70 years old, and 34% and 7% in those ≥75 years old, respectively [36]. In a phase 2 study of nivolumab for previously treated advanced squamous NSCLC [34], any-grade TRAEs were reported in 57.3% of all patients (*n* = 811), 62.9% of patients aged ≥70 years (*n* = 278), and 68.8% of patients aged ≥75 years (*n* = 125). Grade 3/4 TRAEs were reported in 13.9% of all patients, 15.8% of patients aged ≥70 years, and 18.4% of patients aged ≥75 years. Yamaguchi et al. [40] retrospectively examined TRAEs in elderly patients (≥75 years) and analyzed them according to two age groups (75–79 years, *n* = 101 and ≥ 80 years, *n* = 30). Any-grade immune-related adverse events (AEs) were not significantly related to age (38% of all patients, 37% aged ≥74–79 years, and 41% aged ≥80 years). Spigel et al. [33] evaluated TRAEs in a large number of patients (*n* = 1426), although the cut-off age was not 75 but 70 years. The proportions of any-grade and grade 3/4 TRAEs were 62% and 12%, respectively, in patients 67 (range: 23–93) years old and 64% and 14%, respectively, in patients ≥70 years old (*n* = 556) compared with the total population. Thus, most reports have described a similar frequency of AEs induced by ICIs between the elderly and non-elderly. These results suggest that elderly patients ≥75 years old can safely receive ICI monotherapy.

## 8. Conclusions and Future Directions

The molecular diagnosis of advanced NSCLC is considered essential for both elderly and non-elderly patients. Several prospective randomized or non-randomized, retrospective, registrational, insurance-based, and community-based studies have shown that elderly (≥75 years) and non-elderly patients are similarly treated with ICIs effectively and safely. However, patient selection is needed to administer ICIs in the elderly because they are more likely to have a poor PS with comorbidities, which lead to little benefit, even in non-elderly patients [41,42]. In addition, sub-analysis of patients ≥75 years has not shown that ICIs are significantly more effective than chemotherapy alone. A prospective randomized study comparing ICIs with chemotherapy or ICIs plus chemotherapy with chemotherapy alone in advanced NSCLC patients ≥75 years might be needed. In addition, a cost-benefit analysis should be performed because ICIs are more expensive than chemotherapy [43,44]. If molecular biomarkers determining the efficacy of ICIs are established, it would be reasonable to use ICIs similarly molecular-targeted agents. There is a need for more evidence showing the benefit of ICIs in NSCLC patients ≥75 years, who represent nearly 50% of all NSCLC patients and will continue to increase in number. At this point, we could not decide if elderly lung cancer patients aged ≥75 years benefit from ICIs.

## Figures and Tables

**Table 1:** Hazard ratio for overall survival in <75 vs. ≥75 year-old patients with advanced non-small cell lung cancer in phase III studies.

1st Author	Study	Line	Histology	Therapy	Median Age	Subgroup	Patients	Hazard Ratio
Year	Name		(PD-L1 status)		(Range)	Age	Number	(95% CI)
Brahmer	CM017	2nd	Sq	Nivolumab	62 (39–85)	<65	152	0.52 (0.35–0.75)
2015				Docetaxel	65 (42–84)	≥65, <75	91	0.56 (0.34–0.91)
						≥75	29	1.85 (0.76–4.51)
Borghaei	CM057	≥2nd	Non-Sq	Nivolumab	61 (37–84)	<65	339	0.81 (0.62–1.04)
2015				Docetaxel	64 (21–85)	≥65, <75	200	0.63 (0.45–0.89)
						≥75	43	0.90 (0.43–1.87)
Nosaki	KN010	≥2nd	NSCLC	Pembrolizumab	63 (56–69)*	<75	943	0.64 (0.55–0.75)
2019			(PD-L1 ≥1%)	Docetaxel	62 (56–69)*	≥75	90	0.72 (0.43–1.21)
Nosaki	KN024	1st	NSCLC	Pembrolizumab	64.5 (33–90)	<75	260	0.64 (0.42–0.97)
2019			(PD-L1 ≥50%)	Chemotherapy	66.0 (38–85)	≥75	45	0.49 (0.17–1.39)
								
Nosaki	KN042	1st	NSCLC	Pembrolizumab	63 (57–69)*	<75	1145	0.79 (0.68–0.92)
2019			(PD-L1 ≥1%)	Chemotherapy	63 (57–69)*	≥75	129	0.89 (0.59–1.35)
								
Hellmann	CM227	1st	NSCLC	Nivolumab + Ipi	64 (26–87)	<65	611	0.70 (0.58–0.85)
2019				Chemotherapy	64 (29–87)	≥65, <75	442	0.76 (0.61–0.95)
						≥75	113	0.84 (0.55–1.29)
Reck	IM150	1st	Non-Sq	ABCP	63 (31–89)	<65	441	0.78 (0.60–1.00)
2019				BCP	63 (31–90)	≥65, <75	281	0.69 (0.49–0.96)
						≥75, <85	72	0.78 (0.50–1.76)
						≥85	6	NR
Jotte	IM131	1st	Sq	A + CnP	66 (23–83)	<65	326	0.89 (0.68–1.15)
2020				CnP	65 (38–86)	≥65, <75	279	0.84 (0.63–1.13)
						≥75, <85	77	0.74 (0.45–1.23)
						≥85	1	NR

ABCP: atezolizumab + bevacizumab + carboplatin + paclitaxel; A + CnP: atezolizumab + carboplatin + nab-paclitaxel; CnP: carboplatin + nab-paclitaxel; BCP: bevacizumab + carboplatin + paclitaxel; CI: confidence interval; CM: CheckMate; IM: IMpower; Ipi: Ipilimumab; Non-Sq: non-squamous cell non-small cell lung cancer; KN: KEYNOTE; NR: not reported; NSCLC: non-small cell lung cancer; OS: overall survival; Sq: squamous cell lung cancer; * interquartile range.

**Table 2:**
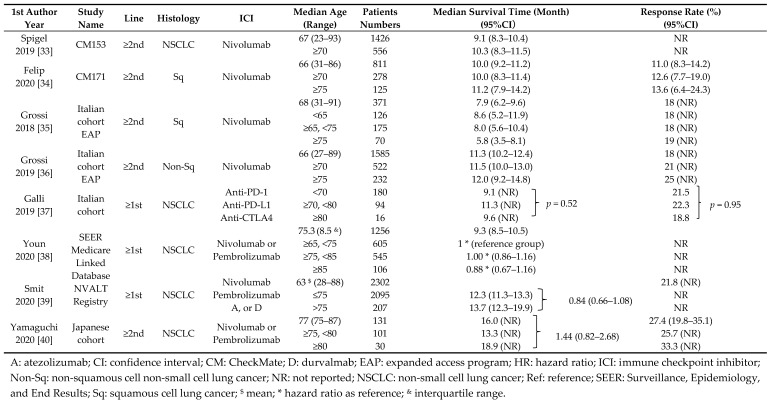
Outcome of elderly non-small cell lung cancer patients treated with immune checkpoints inhibitors in prospective or retrospective studies.

**Table 3:** Adverse events of elderly vs. non-elderly non-small cell lung cancer patients treated with immune checkpoints inhibitors.

1st Author	Median Age	Patients	Treatment-Related Adverse Events (%)	
Year	(Range)	Numbers	Any Grade	Grade 3/4
Grossi	68 (31–91)	371	29	6
2018	<65	126	32	3
	≥65, <75	175	28	9
	75	70	29	3
Grossi	66 (27–89)	1585	33	6
2019	≥70	522	33	7
	≥75	232	34	7
Felip	66 (31–86)	811	58	14
2020	≥70	278	63	16
	≥75	125	69	18
Spigel	67 (23–93)	1426	62	12
2019	≥70	556	64	14
Yamaguchi	77 (75–87)	131	38*	NR
2020	≥75, <80	101	37 *	NR
	≥80	30	41 *	NR

NR: not reported; * Immune-related adverse events.
